# Engineering functional and anthropomorphic models for surgical training in interventional radiology: A state-of-the-art review

**DOI:** 10.1177/09544119221135086

**Published:** 2022-11-15

**Authors:** Zhuo Zhao, Yangmyung Ma, Adeel Mushtaq, Vignesh Radhakrishnan, Yihua Hu, Hongliang Ren, Wenzhan Song, Zion Tsz Ho Tse

**Affiliations:** 1 School of Electrical and Computer Engineering, University of Georgia, Athens, GA, USA; 2 Hull York Medical School, University of York, Heslington, York, UK; 3 School of Engineering and Materials Science, Queen Mary University of London, London; 4 Department of Electronic Engineering, The Chinese University of Hong Kong, Hong Kong, China; 5 Department of Biomedical Engineering, National University of Singapore, Singapore; 6 Department of Computer Science, University of Georgia, Athens, GA, USA

**Keywords:** Anatomical phantom, gelatin, 3D printing, interventional radiology

## Abstract

Training medical students in surgical procedures and evaluating their performance are both necessary steps to ensure the safety and efficacy of surgeries. Traditionally, trainees practiced on live patients, cadavers or animals under the supervision of skilled physicians, but realistic anatomical phantom models have provided a low-cost alternative because of the advance of material technology that mimics multi-layer tissue structures. This setup provides safer and more efficient training. Many research prototypes of phantom models allow rapid in-house prototyping for specific geometries and tissue properties. The gel-based method and 3D printing-based method are two major methods for developing phantom prototypes. This study excluded virtual reality based technologies and focused on physical phantoms, total 189 works published between 2015 and 2020 on anatomical phantom prototypes made for interventional radiology were reviewed in terms of their functions and applications. The phantom prototypes were first categorized based on fabrication methods and then subcategorized based on the organ or body part they simulated; the paper is organized accordingly. Engineering specifications and applications were analyzed and summarized for each study. Finally, current challenges in the development of phantom models and directions for future work were discussed.

## Introduction

The development and widespread application of medical imaging systems has led to significant technological advancements in interventional radiology over the past few decades.^1^ As a result, image-guided, minimally invasive interventional radiology has been on the rise in recent years.^1–5^ It is a low-risk alternative to traditional medical and surgical therapies, offering higher accuracy, fewer complications, and shorter procedural times.^6^ To further improve diagnosis or therapy efficiency based on image-guided interventional radiology, navigation systems, and robotics systems have been developed. Clinical training and education with these new systems are necessary to optimize patient outcomes.^7,8^

Traditionally, training is carried out on live patients, cadavers or animals under the supervision of skilled physicians; however, this approach is expensive, unstructured, has time constraints, and poses risks to patient safety.^8,9^ Alternative training and education models include virtual/haptic-based simulators and anatomical phantoms. Compared to virtual/haptic-based simulators, anatomical phantoms are more popular as they can replicate the external shape and/or physiological characteristics of body parts and organs, modeling the body part closer to its real anatomical structure. In addition, the low-cost anatomical phantom reproduction makes them affordable compared to alternative model methods.^10–12^ Besides medical training, researchers widely use anatomical phantoms to evaluate the performance of new devices and new diagnostic or therapeutic procedures. Radiologists often use phantoms to evaluate radiation doses from imaging devices. There are many cases in which commercial anatomical phantoms are not suitable for the specific requirements of researchers and physicians. For this reason and cost limitations, researchers, and physicians often prefer to develop their phantom prototypes.

This review article focused on the ongoing research in anatomical phantom prototypes developed by researchers and physicians. Gel-based and 3D-printed phantoms were considered, and phantoms of any organ or other body parts were within the paper’s scope. Each phantom was analyzed and summarized for its technical specifications and clinical applications. Finally, in the discussion section, current challenges and directions for future work were presented.

## Methods

Keywords “anatomical phantom,”“phantom for interventional radiology,” and “medical phantom” were searched in PubMed.,^13^ ProQuest.,^14^ Library of the University of Georgia.,^15^ ScienceDirect,^16^ IEEE Xplore,^17^ and Google Scholar^18^ to identify potentially relevant articles. The search range was from January 1, 2015, to June 15, 2020. This search generated an initial pool of 189 articles.

**Figure 1.**
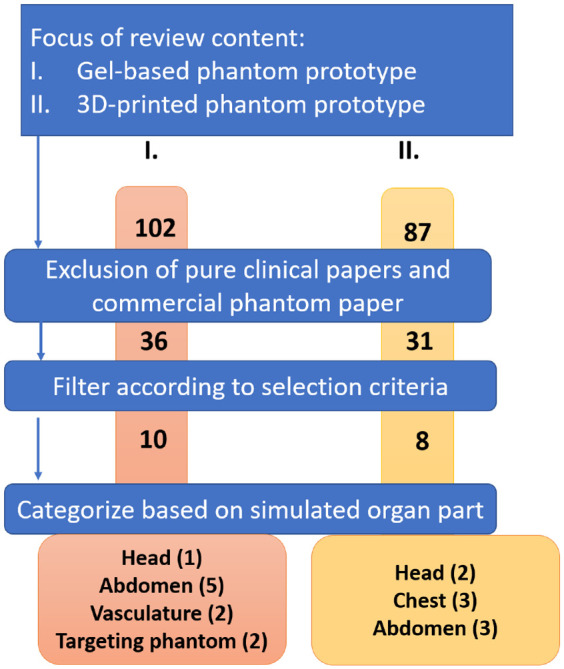
The research process for each category of literature addressed in this article.

The initial pool was evaluated to eliminate irrelevant articles. Firstly, each article abstract was manually analyzed to excluding papers discussing commercially available phantom products and papers that do not discuss the phantom fabrication. After this, a pool of 67 papers was selected. Next, these 67 articles were read in full. Articles on similar research projects and articles that did not report fabrication methods were eliminated. After this step, 18 articles remained (Figure 1).

In the results section, the reviewed phantoms were divided into two categories based on two major fabrication methods: gel-based and 3D printing-based phantom prototypes. Within each category, the phantoms were subcategorized based on the organ or body part they simulated.

## Results

In this section, phantom prototypes developed by gel-based methods (section 3.1) and 3D printing-based methods (section 3.2) are reviewed.

### Gel-based methods

Gel-based phantoms or components to be used along with a phantom have become increasingly popular due to the widely varying properties of gel and the compatibility of gel with ultrasound imaging.^19^ This part of the review discusses the benefits and limitations of gel-based products and their use in aiding interventional radiology training. A summary of the phantoms reviewed in section 3.1 is provided in Table 1.

**Table 1.** Summary of reviewed gel-based phantom prototypes.

Organ part	Phantom name	Materials	Methods	Application	
Head	Head and neck gel-based phantom	Microwave-safe plastic container (2L), Knox gelatin, Psyllium husk fiber powder, green olives, blueberries, food coloring, boiling water, pig laryngotracheal complex	A container holding the gel solution was refrigerated overnight.	Training head and neck surgical procedures involving ultrasound-guided fine-needle aspiration	Richardsonet al.^19^
Abdomen	CT-guided abdominalbiopsy training phantom	Polylactic acid, Formlabs’ flexible material, electromagnetic (EM) tracking coil, super soft plastic, plastic softener, mineral oil (2%). The ratio is 3:1 between soft plastic and softener	3D-printed shell, tumor, and spine. The abdomen was built with a soft plastic mixture solution.	Training in CT-guided abdominal biopsy	Tayloret al.^20^
	Patient-specific ultrasound liver phantom	Silicone from ComposiMold^®^ and Ecoflex^®^, graphite, vaseline oil	Molds were 3D printed. Melted silicone was poured on molds. All molds were degassed before curing. They were assembled using lines to hold the lesions and vessels in place.	Training in diagnostic and interventional procedures	Pacioni et al.^7^
	Liver-mimicking phantom	Polyvinyl alcohol (PVA) (5%), silica gel particles (1%), water, mold (30 cm in length, 15 cm in width, and 16 cm in depth)	Heated PVA mixture solution was poured into a mold, kept at room temperature for 6 h, placed in a freezer at −20°C for 14 h, and then kept at room temperature for 8–10 h.	Compare commercially available needles in terms of their echogenicity in 3D ultrasound images	Arif et al.^21^
	Nephrostomy training phantom	Knox gelatin, metamucil, cinnamon leaf oil, warm water, latex glove, intravenous extension tubing, two tongue depressors, small rubber band, plastic medical tape, Luer lok syringe, disposable suction canister	The opening of a glove filled with water was tightly fastened around intravenous (IV) extension tubing. Then the glove was taped inside a canister filled with the geltain mixture. Finally, it was set in the refrigerator overnight.	Training in nephrostomy techniques	Shamah and May^22^
	Spine phantom	Polyvinyl chloride (PVC) lumbar spine phantom, polystyrene-polyisoprene-polystyrene (SIS) triblock thermoplastic polymer, mineral oil, stainless steel foodservice tray (4-L)	SIS triblock thermoplastic polymer with polystyrene was mixed with mineral oil, placed in a food service tray, and heated up. Then a PVC model was immersed in the mixture and cooled overnight.	Training in fluoroscopically guided lumbar puncture	Faulkneret al.^23^
Vasculature	Blood vessel phantoms	Gypsum, poly(vinyl alcohol) hydrogel (PVA-H), silicone	A reconstructed blood vessel was transferred into a model made of gypsum. The vasculature was suspended in a box-shaped mold. PVA-H was poured into the mold and then the mold was removed. This process was repeated with silicone.	Training on interventional devices for blood vessels and technical assessment of these medical devices	Yu et al.^24^
	Ultrasound phantom for vessel cannulation and targeted biopsy training	Unflavored gelatin powder, hot water (boiling to tepid), one teaspoon of corn starch, unsweetened evaporated milk, Dettol antiseptic liquid, food coloring (blue and red), a calibrated jug with a 1 L capacity for measuring, suitably sized and shaped containers, 22- or 23-G spinal needle, 5 mL syringe, spoon, gauze	Vascular phantom container: Passed the cables through holes on a container and sealed the holes with wax. Swept the cables with oil-soaked cotton piece. Poured the mixed gelatin into the container and kept it at 6°C for 6 h. Removed the wax, fixing the cables. Submersed phantom in water.	Training in vessel cannulation and targeted biopsy procedures	Qurash et al.^25^
		For vascular phantom: Two electric cables about 30 cm in length and 5–10 mm in caliber, wax, and oil-soaked gauze			
		For biopsy phantom: Gelatin, corn starch, and small containers	Biopsy phantom: Mixed different concentrations of corn starch with gelatin and hot water, then kept at 6°C for 2 h to get a target. Placed the target on top, covered it by one-fourth of the mixture, allowed it to congeal (target layer). Then, one-fourth of the mixture was poured over the trapped inclusion and cooled until firm (body layer). Spread the gauze submersed previously in the gelatin mixture and covered with the residual mixture (skin layer).		
Targeting phantom	Polymer-based dosimetry gel tumor phantom	Acrylamide (3%), N,N-methylene-bis-acrylamide (PAGAT) (3%), gelatin Sigma Aldrich (6%), bis[tetrakis(hydroxymethyl)phosphonium] chloride (10 mM), nitrogen, tumor mold, polymethyl-methacrylate (PMMA), water, porcine lung nickel-sulfate (NiSO_4_) (1.25 gL^−1^), water-filled silicon balloon, ultrasound gel, vacuum pump	Gel was injected into a tumor mold. The space between the walls was filled with water, and the inner volume was filled with either water or a lung-equivalent material. A water-filled silicon balloon simulated the diaphragm.	To simulate a gel tumor that has dosimetric validation, which can be used alongside organ-simulating phantoms. For training purposes.	Mann et al.^26^
	Needle-based procedure training phantom	Plexiglass prism of dimensions 220 × 150 × 175 mm^3^, 200 g gelatin, warm water	Conically shaped radiopaque markers were placed at the bottom of a Plexiglass cube. Twenty-eight holes were created in the lid of the phantom. The interior of the cube was filled with gelatin and warm water. The mixture was poured into the cube and cooled to 4°C until it was set.	Training in needle-based treatments such as ablation and brachytherapy	Venturi et al.^27^

Gelatin, silicone, soft plastic, and PVA are the most commonly used materials for building gel-based phantoms. Based on our experience, gelatin and silicone phantoms are easiest to operate. However, it is difficult to control the stiffness of these phantoms, and the use of needles on them can leave visible tracks. The stiffness of soft plastic and PVA phantoms is easier to control in comparison. With soft plastic, any stiffness can be easily acquired by adjusting the ratio between plastic and softness, so soft plastic is suitable for building phantoms that require different regions to have different stiffnesses. Compared to soft plastic phantoms, PVA phantoms tend to be smoother and more durable.

#### Head

**Figure 2.**
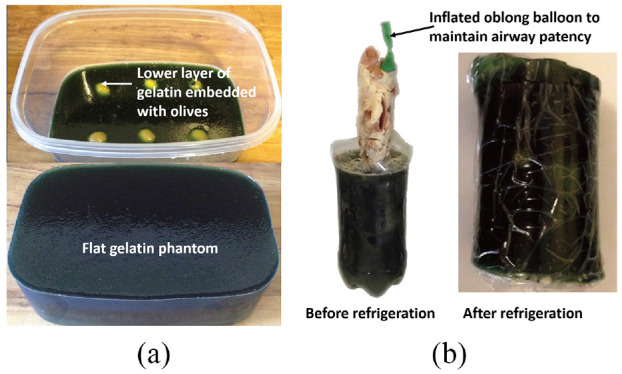
(a) Gelatin-based flat phantom for ultrasound-guided fine-needle aspiration procedures.^19^ and(b) Gelatin-based cylindrical phantom with the addition of a pig laryngotracheal complex for ultrasound-guided fine-needle aspiration procedures.^19^

Richardson et al.^19^ designed two gelatin-based phantoms with the purpose of training head and neck surgical procedures involving ultrasound-guided fine-needle aspiration. One was a flat phantom (Figure 2(a)) for basic techniques and one was a cylindrical phantom (Figure 2(b)) containing a pig laryngotracheal complex for more advanced techniques. Knox gelatin, water, and psyllium husk powder were used to form the gel. Different gelatin layers were achieved by changing the ratio of water and psyllium to the gelatin to simulate the varying levels of tissue echogenicity. Olives and blueberries were used to simulate nodules in the two models. Once the models were complete, fine-needle aspiration techniques were practiced using a Sparq diagnostic ultrasound system. Richardson reported that both phantoms enabled users to acquire ultrasound images and carry out fine-needle aspiration techniques, with the fabrication cost of each model amounting to less than $40.

#### Abdomen

**Figure 3.**
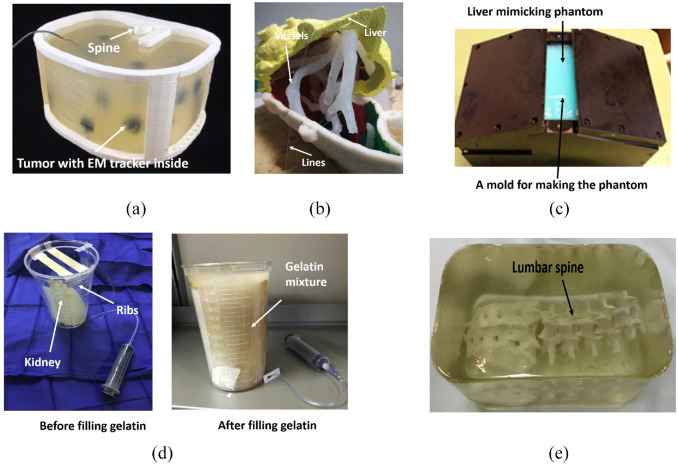
(a) The developed abdominal phantom,^20^(b) Phantom liver assembly. The vessels and the lesions have been held in the correct position by means of lines,^7^ (c) The mold used to construct liver-mimicking phantom from PVA,^21^ (d) The $5 nephrostomy training phantom,^22^ and (e) The developed lumbar puncture training phantom.^23^

Taylor et al.^20^ designed an abdominal phantom (Figure 3(a)) for needle insertion practice. The phantom and phantom lesions’ shell was designed using CAD and fabricated via 3D printing, and an electromagnetic tracking coil was inserted into the phantom. A spine phantom was produced via CAD and 3D printing using polylactic acid, which was also used to print the shell and inserted into the abdominal phantom. The abdominal phantom was filled with gelatin material, a mixture of soft plastic and plastic softener containing 2% mineral oil. A mixture ratio of 3:1 between soft plastic and plastic softener was selected after rigorous testing to obtain a similar density and penetrability compared to the human abdomen. The lesions were placed at various heights of the phantom by constructing the inside filling of a phantom in four layers. The total cost amounted to approximately $57. CT scans showed clear visibility of the filling, lesions, and tracking coil. Hence, the locations of the lesions and the needle tip were successfully tracked based on CT scans and OncoNav software. Furthermore, an average lesion targeting success rate of 76.9% was achieved by five medical trainees.

Pacioni et al.^7^ developed a training phantom for liver ultrasound (Figure 3(b)). To make the phantom, CT images of real patients were processed, and 3D models were extracted and printed from acrylonitrile butadiene styrene. To create vessels and lesions in the liver phantom, various silicone mixtures were used. The liver parenchyma was made of 53% silicone, 5% graphite (as an echogenicity enhancer), 15% thinner, 20% Vaseline oil, and 7% slacker. The hypoechoic lesion contained 88% silicone, 2% graphite, and 10% Vaseline oil. Hyperechoic lesions were made from silicone dipped in Vaseline oil. Pure silicone was used to construct anechoic lesions, vena cava, and portal vein. Each liver phantom costs around $100, much less than the $300 industry-standard CIRS phantom. Among the 15 physicians who used the low-cost liver phantom, all agreed that it was either the same or better than commercial phantoms in 16 categories that addressed the phantoms’ real properties. Overall, it was agreed that the phantom reproduced the human liver’s morphology well and allowed vessels and lesions to be seen in ultrasound images. Although the sound speed in the phantom was not true to the liver, the study acknowledged that this could be overcome with a software adjustment.

Arif et al.^21^ used a liver-mimicking phantom (Figure 3(c)) to test the visibility of various needle tips in 3D ultrasound images. A 5% aqueous solution of polyvinyl alcohol was the main material used to construct the phantom. Ultrasound scatters simulated with silica gel particles (1%). The mixture was heated, placed into a mold, and then left at room temperature. After rest, it was frozen at −20°C and kept at room temperature. This process was repeated two times to increase the stiffness of the model. The phantom was used to compare the visibility of seven different needles between phantom and cow liver to help decide what to use for training. The test results indicated that better visibility could be achieved in the phantom than in the cow liver.

Shamah and May^22^ devised low-cost ($4.48 per phantom), easy-to-make, and uncomplicated nephrostomy phantoms (Figure 3(d)) using common household items and hospital supplies for ultrasound-guided percutaneous nephrostomy procedures. The gel for this phantom was made by mixing Knox gelatin and tap water. In the mixing procedure, cinnamon oil was used to eliminate air bubbles, and Metamucil was applied to add echotexture to the solution. Metamucil also made the gelatin opaque, which was not conducive to needle navigation. The kidney in the phantom was simulated using a small latex glove, with its tied fingers serving as the blunted, hydronephrotic calyces. The gel caused a certain degree of flexibility, so tongue depressors were added to simulate the ribs. After the kidney was made, the canister holding the kidney was filled with gelatin and placed in the refrigerator to set. This way, the phantom was produced within 1 h and could be stored for up to 2 weeks. Tests conducted by Shamah and May showed that the phantom could withstand multiple punctures, enabling trainees to target multiple calyces from different angles and test out different ultrasound probe positions.

Accurate lumbar spine phantoms currently available on the market are rarely used for training in fluoroscopy-guided procedures since these phantoms are expensive and unable to mimic the appearance of the bone and soft tissue in fluoroscopic images. Therefore, Faulkner et al.^23^ constructed an inexpensive and durable lumbar spine phantom made entirely of gel (Figure 3(e)) for fluoroscopy-guided lumbar puncture training. Injection molding of polyvinyl chloride (PVC) was used to fabricate the part of the phantom simulating bone because PVC is inexpensive, has a similar density to the bone, and tolerates high temperatures to 140°C. Polystyrene-polyisoprene-polystyrene triblock thermoplastic polymer mixed with mineral oil was used to fabricate the part of the phantom simulating soft tissue. This mixture was poured into a steel food tray, heated up, and stirred before the spine model was submerged in the mixture and allowed to cool. Creating the phantom cost approximately $148 and took 10 h to make in total. The conducted test showed the phantom was found to be fluoroscopically compatible, robust, could be heat-treated to repair needle track marks and cost-effective due to its ability to be indefinitely reused.

#### Vasculature

**Figure 4.**
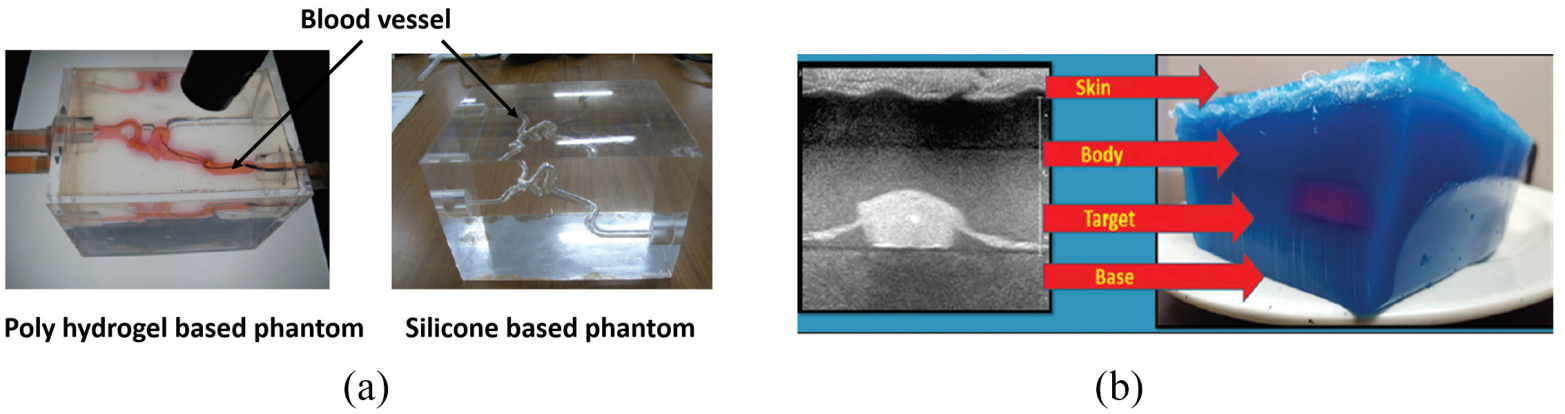
(a) The box-shaped PVA-H phantom model and silicone phantom model.^24^ and (b) Cross section of the biopsy phantom showing its layers and corresponding ultrasound appearance.^25^

In order to investigate the traceability of interventional devices, Yu et al.^24^ created blood vessel phantoms composed of poly(vinyl alcohol) hydrogel (PVA-H) and silicone (Figure 4(a)).This study was carried out to increase the number of endovascular treatments implementing catheters.^28,29^ The goal for the blood vessel phantoms was to simulate real-world conditions better to provide more accurate interventional training and allow for the technical assessment of medical devices.^30^ The creation of the phantom began with a 3D model of a patient’s cerebral vasculature. This 3D geometry was then replicated in a model made of gypsum, and this bio-model was placed in a box-shaped container into which PVA-H was then poured to create a mold. PVA-H is made up mainly of poly(vinyl alcohol) and water, and it forms a hydrogel in high temperatures. Freeze-thawing was used to endow the gel with high strength,^31^ but it also lowered the hydrogel’s transparency, which was solved by using dimethyl sulfoxide aqueous solution as the solvent. This resulted in a gel that was high in both transparency and tensile strength. This phantom model could be replicated using silicone. The transparency of silicone allowed for clear observation of the catheter, balloon, and guidewire movements. However, the effectiveness of the interventional device was different in the two phantom models. The higher friction coefficient of silicone and stiffness were the reason cause the difference. Overall, the authors considered both models to be reasonable materials to measure catheter performance and provide accurate surgical training.

Qurash et al.^25^ set out to make two gelatin phantom designs for basic training in vessel cannulation and targeted biopsy (Figure 4(b)). The gelatin mixture was made from water, unflavored gelatin powder, cornstarch, unsweetened evaporated milk, Dettol antiseptic liquid, and food coloring. All flavored or sweetened materials helped gelatin fermentation but also shortened the phantom life. With these ingredients, a vascular phantom and a biopsy phantom were created by pouring the mixture into containers of the desired shape. The study found that its described formula and construction technique offered a phantom that was isoechoic to human tissue, inexpensive (<$10), eliminated needle pass artifacts, gave different levels of opacity for different training levels, vessel calibers, target sizes, and levels of echogenicity, provided tactile feedback, was stable at room temperature for 6 h and offered valid use for up to 2 months if preserved in the refrigerator. The cornstarch helped adjust the echogenicity of the gelatin mixture and controlled the echogenicity of the target with different echo levels as needed and with a significant reduction in the posterior shadowing from other typically used targets.

#### Targeting phantoms

**Figure 5.**
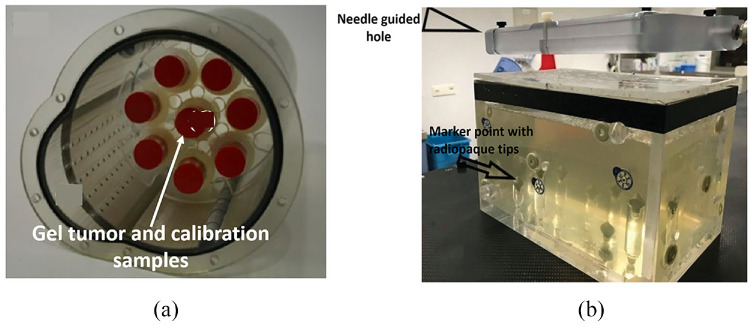
(a) Dosimetry gel-based lung tumor.^26^ and (b) The gelatin-filled plastic phantom with the frame that held the plates containing the needle guide holes.^27^

Mann et al.^26^ developed a gel tumor (Figure 5(a)) to validate 3D dosimetry of the motion compensation concepts in radiotherapy. PAGAT (polyacrylamide gelatin gel fabricated at atmospheric conditions) dosimetry gel was used to simulate a lung tumor.^32^ A suitable method to measure 3D dose distribution was to use polymer gel dosimetry.^33–36^ The 3D polymer gel dosimetry was investigated in terms of its accuracy and feasibility. The gel was calibrated with eight flasks, and one of the eight flasks acted as the gel tumor. The gel tumor was inserted into the lung phantom and irradiated either before or after irradiation of the calibration samples with doses from 0 to 7 Gy. The conducted magnetic resonance evaluation of the 3D dose distribution indicated that the developed phantom offered good results.

Venturi et al.^27^ proposed using phantoms (Figure 5(b)) to evaluate the in vitro accuracy of a new, inexpensive system called ArciNav, which helps guide targeted-in-gantry needle-based procedures. The phantom used in this study was a plexiglass prism with dimensions of 220 × 150 × 175 mm. Eight conical radiopaque markers were distributed at different heights along the bottom of the prism. Twenty-seven holes, each 8 mm in diameter, were randomly distributed across the lid of the phantom, serving as needle entrance points. The locations of the holes could be changed by flipping and rotating the lid. Gelatin was used to mimic the homogeneity of the internal organ by filling the interior of the cube. Gelatin and water were mixed and poured into the cube, and cooled. When the ArciNav system was used on the phantom, its accuracy was similar to other image-guided intervention systems on the market. The study addressed that a limitation was that a wire could slip when the gelatin-filled phantom was moved. The study also reported cases in which the needle was blocked once it touched the target.

### 3D printing method

Individual variance and complexity of the human body make 3D printing the ideal method for manufacturing anatomically accurate, patient-specific models. The rapid printing times, high customizability, and low cost make 3D printing especially useful in forming models for surgical planning and simulation training where information from MRI or CT scans can build the model.^37^ In this section, phantoms developed via 3D printing are reviewed. A summary of this section is shown in Table 2.

**Table 2.** Summary of reviewed 3D-printed phantom prototypes.

Organ part	Phantom name	Materials	Build methods	Applications	Ref.
Head	Apical granuloma phantom with periodontal ligaments	MED690 photopolymer, cavities filled with SUP705	Conventional impressions alongside CT data	Simulation of root tip resection for dental students	Hanisch et al.^38^
	Head phantom	Photo-cured acrylic resin material (for both the head phantom and eye inserts)	Eye insert: Previous eye models were utilized. Head phantom: HRCT image of a human skull embedded in PMMA	Forming task-specific phantoms for dosimetry purposes	Homolka et al.^39^
Chest	Multi-layer breast phantom	Interior containers made from composite plastic. Silicon composite-based skin. Individual chambers can be filled with any material to mimic the breast density of the desired patient.	The breast’s anatomy was simplified into four compartments using anatomic representations and CT scans, of which four containers were 3D printed.	More realistic construction of breast phantoms	Faenger et al.^40^
	Lung airway phantom	Transparent photopolymer resin	Real patient data were used to construct a 3D airway model. This was modified to ensure the entire structure was hollow and subsequently 3D printed in three parts.	Training on medical tools used for image-guided lung intervention	Zhao et al.^41^
	Phantom trachea with main bronchi	Lung parenchyma: Tango™ photopolymer. Cartilage rings: Mixture of photopolymers (Vero Color Blue and Tango™) Stricture: Similar mixture of photopolymers	CT, ultrasonography, and endoscopy medical images of normal anatomy were utilized to segment and subsequently printed the main airway	Determine optimal contrast agent contrast/saline ratio for minimized risk in balloon dilatation	Kim et al.^42^
Abdomen	JAAA (juxta-renal abdominal aortic aneurysm) phantom	Aortic wall made from FullCure 930 TangoPlus photopolymer. Thrombus chamber filled with ultrasound gel.	Diagnostic CTA imaging of FEVAR suitable patient used for the segmentation of JAAA. Modifications made to STL file to allow proper behavior of demonstration FEVAR graft. Phantom printed in two parts and joined by silicon gel.	X-ray guided vascular simulation of JAAA repair	Meess et al.^43^
	Biliary tree phantom	Red acrylonitrile butadiene styrene plastic	An MRCP dataset of a patient with distal common bile duct cholangiocarcinoma with dilated bile ducts was used to form the 3D STL file. Support cylinders were made, and the biliary lumen was hollowed for subsequent printing.	Endoscopic biliary intervention training	Bundy et al.^44^
	Radiopaque abdominal phantom	CT image-based model printed by using aqueous potassium iodide solution. Images separated by polyethylene. Entire phantomcovered by plastic film.	Abdominal CT dataset images were printed to scale with the original patient size on paper using radiopaque potassium iodide. Each image was separated by a 1 mm layer of polyethylene.	Simulation of CT-guided percutaneous procedures	Jahnke et al.^45^

#### Head

**Figure 6.**
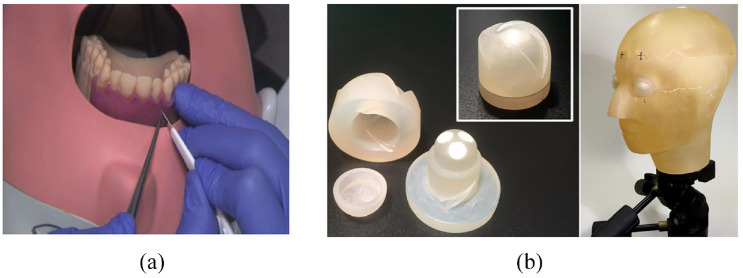
(a) The exercise with the 3D-printed training model in surgical simulation of apicoectomy.^38^ and (b) Photograph of eye insert (left) and head phantom (right) with eye inserts in place mounted on tripod.^39^

Hanisch et al.^38^ studied the feasibility of using 3D-printed phantoms (Figure 6(a)) in surgical simulations of apicoectomy (root tip resection) for dental students. Their production technique consisted of fabricating a plaster cast from a patient, which was trimmed and 3D scanned. Cone-beam computed tomography (CBCT) information from another patient was used to form teeth 11, 12, and 21. The final 3D model was then modified to include periodontal ligaments (which are excluded in typodont models) and a granuloma of the root of tooth 11. The 3D model was printed with an Objet Eden360V printer (Stratasys, Rehovot, Israel), which employed the PolyJet technique. The investigation showed that dental students significantly benefited from using the 3D phantom, and it was not inferior to industrially manufactured training models.

The use of 3D-printed phantoms can extend beyond education and training. Homolka et al.^39^ utilized 3D printing to fashion a head phantom with separate eye inserts (Figure 6(b)) for use in dosimetry studies and compared the phantom with the current gold standard model on the market (i.e. 3M Lucite and RANDO). The aim was to simulate secondary scatter radiation to predict specific organ exposure to radiation better. To form the eye inserts, minor adjustments were made to pre-existing eye models^46^ to ensure that they could be smoothly inserted into the head phantom. Each eye was printed in three parts to allow for insertion of the three TL dosimeter chips (GR 200A, Solid Dosimetric Detector and Method laboratory, Beijing, China) and was printed using a Solid Edge ST5 3D printer (Siemens PLM Software, Plano, TX, USA). The head phantom used a HRCT dataset of a 3M Lucite skull phantom (3M, St. Paul, MN, USA) embedded in PMMA. The STL mesh resulting from the CT scan reconstruction was segmented to identify the air-filled cavities in the skull (frontal, maxillary, and sphenoidal sinuses, and airways) and remove them. The brain volume and surrounding tissue were removed and replaced post-print with deionized water. The conversion, segmentation, post-processing, and optimization procedures were done with Analyze 11.0 (Biomedical Image Resource, Mayo Clinic, Rochester, MN, USA) and Meshlab V1.3.2. (Visual Computing Lab ISTI-CNR, Pisa, Italy). The final print was done using Objet Eden350V (Stratasys, Edina, MN, USA). The phantom’s hollow regions were filled with supporting material, which was mechanically and chemically cleared using sodium hydroxide solution with a water jet system. The dosimetry studies showed that the printed phantom recorded a 15% higher dose on the eye when the dose was directed to the forehead, which is also shown from the RANDO phantom. Due to constraints on materials that can be used, it was concluded that the printed phantom could not represent an ideal model for dosimetry studies.

#### Chest

**Figure 7.**
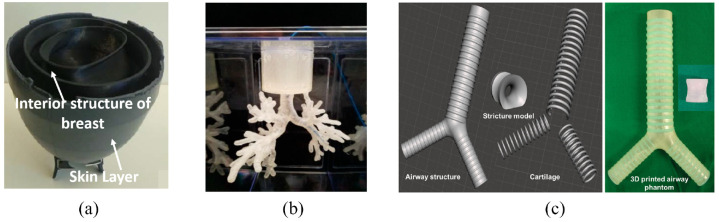
(a) 3D-printed breast phantom,^40^ (b) 3D-printed lung airway phantom for training and evaluation purposes,^41^ and (c) The images show the 3D design of the simulation models (left) and a 3D printed phantom (right) of the trachea, including an airway structure, cartilage, and a stricture. The trachea was 21 mm in diameter, and the stricture part was 10 mm in diameter.^42^

There has been recent interest in using microwave imaging within medicine to exploit the differences in dielectric properties between malignant and non-malignant breast tissue for earlier diagnosis.^47^ As a result, Faenger et al.^40^ designed a customizable 3D-printed breast phantom (Figure 7(a)) to evaluate these systems. The breast’s anatomy, modeled using CT information and anatomical representations, was divided into four main chambers: mammary glandular tissue, lactiferous ducts, adipose tissue, and skin. The phantom was designed using Inventor 2017 (Autodesk, San Rafael, CA) to allow for the generation of asymmetric structures. It consisted of two interior chambers made from both 3D-Prima Conductive ABS and Proto-Pasta Conductive PLA filaments, as they have superior dielectric and printing properties compared to pure plastic. The chambers could be filled with liquid to mimic mammary gland tissue or lactiferous ducts according to variability in breast anatomy. Furthermore, the open-top design allowed for the integration of structures mimicking tumors. The skin covering the phantom was made from a silicon composite material to mimic the skin’s dielectric properties better.

Zhao et al.^41^ investigated the performance of a 3D-printed endobronchial phantom (Figure 7(b)) in simulating electromagnetic (EM) tracking and ablation procedures. A 3D dataset from a scanned airways model, sourced from the National Institutes of Health (NIH), was imported into Meshmixer (Autodesk, San Rafael, CA) for rebuilding. Once rebuilt, the structure was hollowed to form the lumen of the lung airways, and the separate shell function was used to simplify the model for printing and ensure the lumen was large enough for instrumentation. The final printing process was carried out using two different machines for comparison: a Lulzbot 3D printer (Aleph Objects, Inc, Loveland, Co, USA) and a Form 2 3D printer (Formlabs, Cambridge, MA, USA). The Form 2 phantom was more expensive to manufacture ($29.80 vs $15.6 USD) but took less time to print (30 vs 40 h) and had a much higher resolution (0.05 vs 0.2 mm). To evaluate each model’s performance, CT images were acquired and reconstructed by OncoNav (NextPath, Wall Township, NJ, USA) for subsequent registering. A custom EM tracking catheter was used to navigate the phantom, and the distance between the guided and tracking point was used to measure accuracy. An ablation test was done using thermochromic gel to act as an indicator of correct placement and heating of the area. Results showed that both phantoms could be used for testing new lung interventions at a low cost.

Kim et al.^42^ utilized medical CT, ultrasonography, and endoscopic datasets in patients with normal anatomy, alongside a human anatomy atlas, to deduce the human’s average morphological information trachea and main bronchi for 3D printing (Figure 7(c)). This was done to investigate whether the contrast agent dilution affected balloon deflation times during tracheal dilatation procedures. The airway model was simulated using MeshLab (Visual Computing Lab ISTI-CNR, Pisa Italy) and MeshMixer (Autodesk, San Rafael, CA). The final model included three separate parts: the airway parenchyma, cartilaginous tissue, and a stricture. The parts were printed using a Connex3 Objet500 (Stratasys Corporation, Edina, MN, USA). The airway tissue was made from a rubber-like material from the Tango™ family; the cartilage was made from a 70:30 ratio of the same rubber material and Vero Color Blue; and the stricture was made from a 65:35 ratio of the same materials as the cartilage, except Vero Color Red was used instead of Vero Color Blue. Using this phantom, it was found that utilizing a more dilute contrast agent allowed for decreased deflation time and subsequently limited potential end-organ ischemia.

#### Abdomen

**Figure 8.**
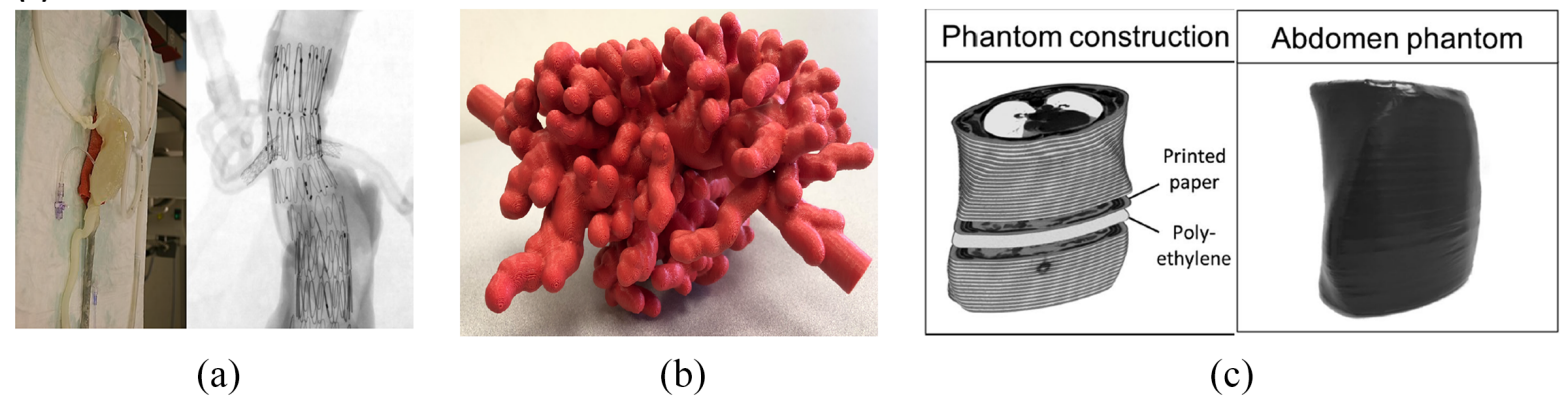
(a) Illustration of the final 3D-printed phantom of the patient abdominal aortic aneurysm,^43^ (b) External appearance of 3D-printed biliary endoscopy phantom,^44^ and(c) 3D-printed abdomen phantom.^45^

Meess et al.^43^ utilized 3D printing to fabricate an abdominal aortic aneurysm (AAA) phantom (Figure 8(a)) from FullCore 930 TangoPlus using computed tomography angiography (CTA) information from a patient deemed suitable for a fenestrated endovascular aortic repair (FEVAR) procedure. This specific procedure is deemed technically difficult due to the complex deployment process and involvement of the renal and superior mesenteric arteries in the aneurysm. The cost of the 3D-printed phantom was $254.49, and the printing time was around 13 h. The phantom was then subsequently used for simulation training using a flow apparatus to simulate cardiac pumping. It was found that by simulating the procedure beforehand, potential procedural complications could be predicted and accounted for in the final patient procedure.

Bundy et al.^44^ explored the use of 3D-printed models (Figure 8(b)) for training in biliary tree procedures. An MRCP dataset of a patient with a distal cholangiocarcinoma was selected, and Vitrea (Vital Images, Minnetonka, MN) was used to reconstruct the 3D model from the original dataset. Then, MeshMixer (Autodesk, San Rafael, CA) was used to optimize the model further to create a hollowed model. Finally, 125% scale was applied to print the model to ensure easy access for the endoscope. Printing was done with a Stratasys Dimension Elite Plus printer (Stratasys, Eden Prairie, MN) using acrylonitrile butadiene styrene plastic. In the evaluation procedure, 11 technologists, medical students, residents, fellows, and physicians were asked to use the phantom and, on a 10-point Likert scale, score the likelihood that they would choose to use endoscopy for various procedures before and after the endoscopic training session. It was shown that, after endoscopy training, trainees felt they would be more likely to opt to use endoscopy for gastrostomy, with the likelihood score increasing by 40%. Additionally, the comfort level for using endoscopy to perform cholecystostomy increased by 38.9%. The low cost of the phantom ($172.43) means that this model would fit many budgets, and the phantom can be customized to incorporate different pathologies or anatomical variations.

Although 3D printing is an excellent tool for representing the gross anatomy of the body, it is relatively poor in representing the radiological appearance of structures since the contrast is derived from physical tissue properties. To better simulate CT-guided percutaneous interventions using phantoms, Jahnke et al.^45^ constructed an abdominal phantom (Figure 8(c)) by printing individual CT images in aqueous potassium iodide solution (1 g/mL) using a standard inkjet printer (HP Deskjet 6940; Hewlett Packard, Palo Alto, CA). Each consecutive CT image was separated by a layer of 1 mm thick polyethylene foam cut into the shape of the patient, as shown in Figure 8. The final phantom was covered with a film of black plastic. The performance of the phantom was evaluated by participating interventionalists, who concluded that it was suitable for trainees to learn how to operate CT devices.

## Discussion

For the purpose of training, phantoms are useful in updating the knowledge and skill of trainees and in reducing risks to patients. Although 3D printing-based and gelatin-based building methods can provide low-cost phantoms for researchers and healthcare workers, challenges remain in this field.

One of the disadvantages of utilizing gelatin phantoms is that they are somewhat time-consuming to manufacture, resulting in increased labor costs. The difficulty in controlling the homogeneity of gelatin is another shortcoming. By developing a programmable gelatin injector, the fabrication time could be decreased and the gelatin homogeneity could be controlled easily. However, with a programmable gelatin injector, the preparation time may be increased since it would require detailed model design for programing before fabrication. Furthermore, limitations of gelatin phantoms have been reported.^19^ For example, they can only withstand destructive tasks like biopsies for so long before the simulation quality and efficiency decrease significantly.^7^ This may be solved by creating gelatin with a self-healing ability. In addition, how to make complex phantoms, such as a patient-specific and mimic phantoms with multiple organs and body parts, from gelatin is another big challenge due to the narrow range of available gelatin materials and the difficulty of controlling the homogeneous and inhomogeneous distribution of gelatin.

Compared with gelatin injection, 3D printing is faster and makes it easier to build more complex patient-specific and mimic phantoms. Additionally, more materials can be used by 3D printers, even in one printing procedure, and 3D printers provide stable control of the homogeneous and inhomogeneous distribution of material. However, 3D-printed models need to be segmented from medical images or designed using computer-aided design tools first, and the material properties need to be set in detail in each segmentation to acquire more realistic phantoms. This can be a difficult and time-consuming process, and it increases the expertise needed to construct patient-specific phantoms. These factors would likely cause a significant increase in the labor costs associated with 3D-printed phantoms compared to other phantoms. Building an open community and allowing designers to share different anatomical models could be helpful. Integrating segmentation and rapid design functions into 3D printer software could be another way to decrease the difficulty level. Another obstacle for 3D-printed phantoms is that certain tissue properties cannot as of yet be achieved with low-cost materials.

Integrating realistic tactile stimulation or multiple tissue levels is also a challenge for developing custom-made phantoms. Tactile stimulation helps trainees receive feedback when practicing the procedure of pushing instruments into a patient’s body, such as needle puncture procedures.^23^ Building a phantom with multiple tissue levels where each level is very similar to its corresponding body part could provide tactile feedback, but this approach would be expensive, time-consuming, and labor-intensive even with a 3D printer. Implementing sensors into the phantom during the building process to provide tactile simulation would be more feasible. In this solution, micro position sensors could be implemented into different tissue levels based on advanced planning during phantom design. Users could then wear a haptic glove to receive the signals from these sensors, which would simulate tactile feedback from different tissues. However, this solution’s implementation would still be time-consuming and labor-intensive and increase phantom material and labor costs.

Like the lack of tactile stimulation, the lack of motion simulation in most phantoms is also a challenge. Patient movement or respiration can lead to movements of tissues or organs during surgery. A static phantom cannot provide trainees with a completely realistic portrayal of a surgical procedure, potentially leaving trainees underprepared. Pumps are used to simulate respiration for many lung phantoms, but this may not be a feasible solution for all phantoms. Vibration machines could offer a potential solution, but more methods to control and implement vibration machines need to be explored. Alternatively, some simple mechanical setups have been reported to simulate respiratory motion in liver phantoms^48^ effectively. These methods could also be a potential direction to simulate the motion in phantoms, modeling other anatomical structures.

## Conclusions

Training and evaluating medical students in surgical procedures is vital for ensuring the safety and efficacy of surgery. In recent years, anatomical phantoms have become more and more popular for training medical students rather than relying on traditional training on real patients. The benefits are that anatomical phantoms eliminate risks to patients and offer more efficient training. Although some anatomical phantoms are commercially available, researchers and physicians often have to develop their phantom prototypes in situations where specific needs and functions are required and the budget is limited. In this article, researchers and physicians developed phantom prototypes based on two major methods – the gel-based method and the 3D printing-based method – which were reviewed. Much of the literature mentioned in this paper focused on developing medical phantom prototypes for interventional radiology training. Ultimately, 18 related papers were selected for review. And this paper focuses on the engineering aspects of the Functional and Anthropomorphic Models and therefore the phantom’s utility and user feedback are not involved in the scope of this review. Although gelatin and 3D printing are frequently used to develop phantoms, both have some limitations, such as being time-consuming and labor-intensive and lacking low-cost materials to simulate tissues and organs properly. In the future, 3D printing could be an efficient way to develop patient-specific phantoms for training and evaluation purposes. However, to apply it widely, a simplified molding process and more low-cost printable materials that closely mimic tissues and organs are necessary.
